# Supplementary material for: Pathophysiological Mechanisms of Cardiac Dysfunction in Transgenic Mice with Viral Myocarditis
Source: Cells. 2023 Feb 8;12(4):550. doi: 10.3390/cells12040550 (PMC9954433; doi:10.3390/cells12040550)
Supplement: Supplementary file 1 [file cells-12-00550-s001.zip › cells-2129888-supplementary.pdf]

Supplementary material

Table S1. Progressive dilation of murine cardiomyocytes.

Age	25 ± 2 Weeks	30 - 45 Weeks	46 - 54 Weeks
Length (μm)	149.4 ± 2.20	157.3 ± 1.19	163.9 ± 1.47
Length (%)	100 ± 1.47	105.3 ± 0.80	109.7 ± 0.98
Area (μm^2)	3709 ± 99.72	4023 ± 52.08	4482 ± 66.55
Area (%)	100 ± 2.69	108.4 ± 1.40	120.8 ± 1.79

Values are given as mean ± SEM of the respective morphological value at the indicated age (n = 239–791).

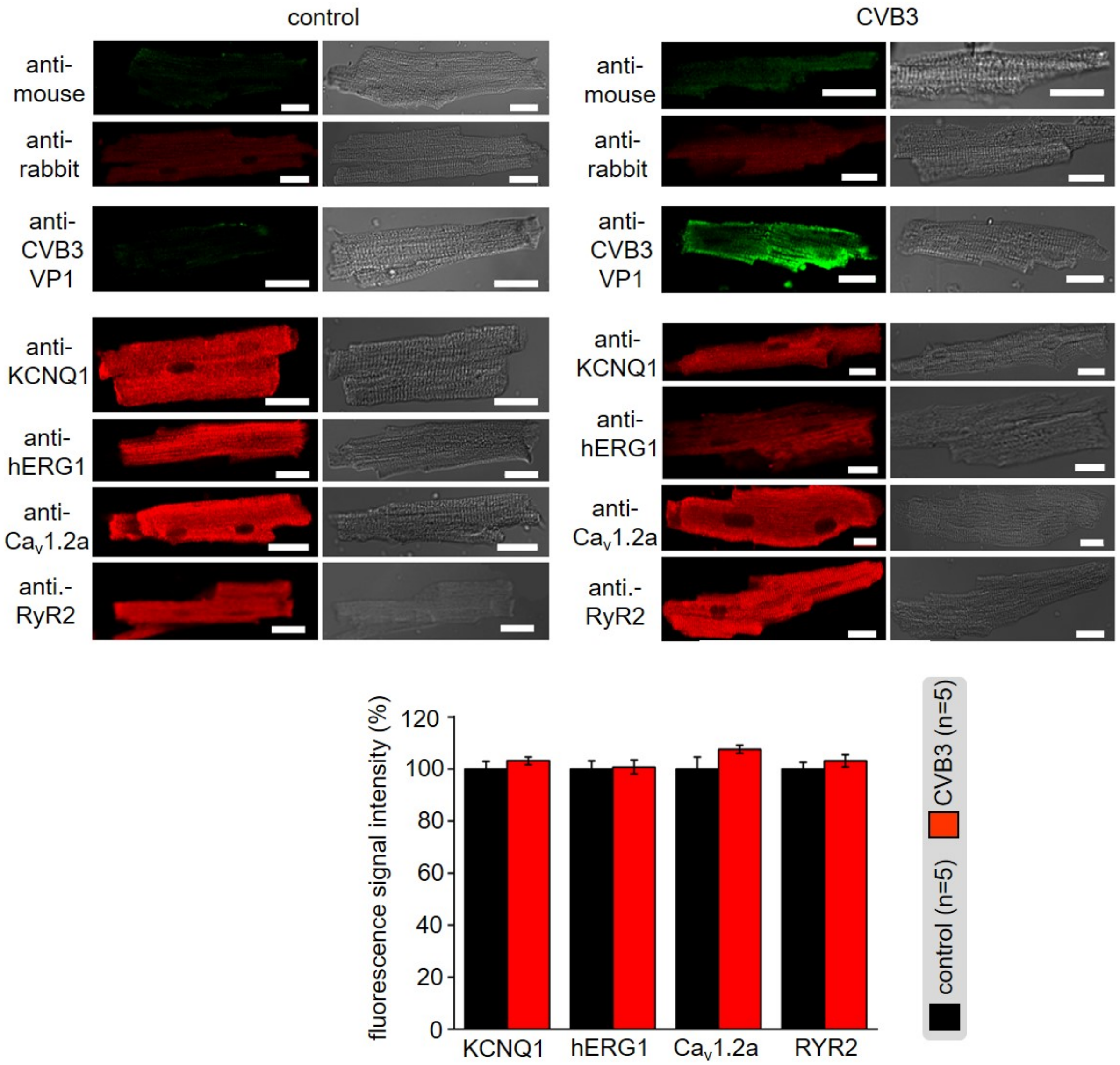

Supplementary Figure S1: Exemplary immunocytochemical staining of fixed cardiomyocytes isolated from wild-type littermate (left panel) and a transgenic (right panel) mouse. The control stains anti-Rabbit and anti-Mouse served as reference for the images of the respective colour channel. As further verification of the genotype, a fluorescent labeling of the viral CVB3 capsid protein VP1 was performed, which is clearly visible here in the CVB3-positive mouse cell (right). The transmitted light images show, due to the fixation method, a granulation of the cell surface similar to the control cells (scale bar 20 m). The labelling of the ryanodine

receptor was used in later analysis to create a fluorescence profile for a distance measurement of the T-tubules in both investigated populations. Red: AlexaFluor 594; green: AlexaFluor 488. The labelling of the cardiac ion channels KCNQ1, hERG1 and Ca$_v$1.2 verifies the cardiac cell identity. Quantification of the fluorescence signal intensities of the labelled ion channels shows no differences between wt littermates and CVBΔVP0-expressing cells

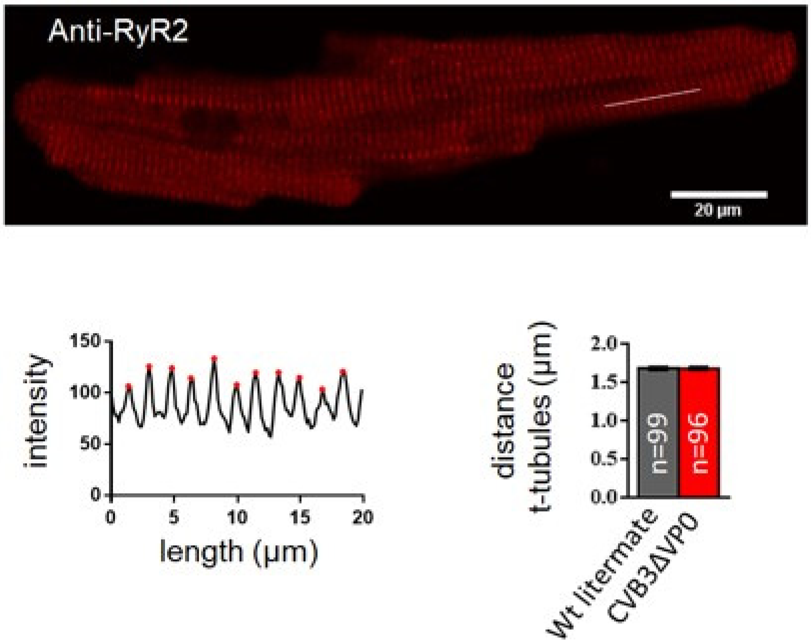

Supplementary Figure S2: Exemplary immunocytochemical staining of of the ryanodine receptor in fixed cardiomyocytes indicate the distance of t-tubules. The labelling of the ryanodine receptors in wild-type littermate and a transgenic mouse similar as in suppl. Figure 1 was used in later analysis to create a fluorescence profile for a distance measurement of the T-tubules in both investigated populations (example lower left). The distances of the thus analysed t-tubules are virtually identical in wt littermates and transgenic mouse cardiomyocytes.

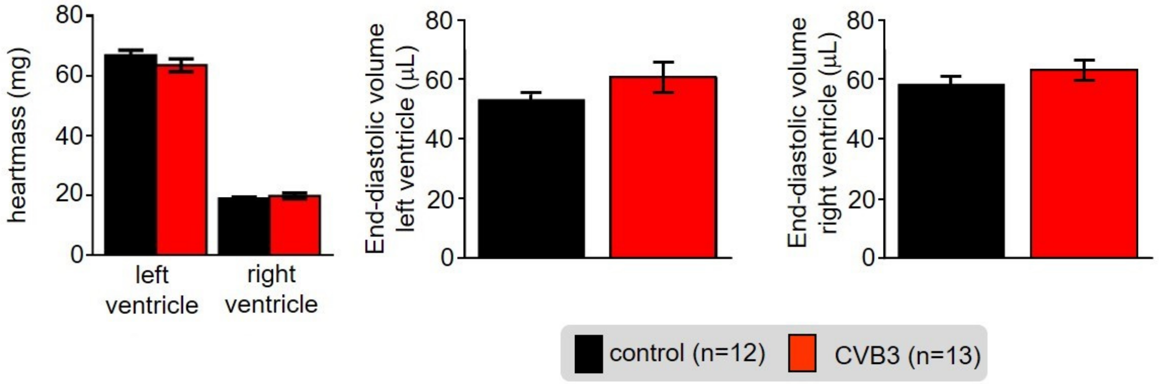

Supplementary Figure S3: heartmass, and end-diastolic volumes of left and right ventricles measured in control and CVB3-expressing mice. No significant changes were observed in the illustarted parameters.
